# *In Vitro* High Throughput Screening, What Next? Lessons from the Screening for Aurora Kinase Inhibitors

**DOI:** 10.3390/biology3010167

**Published:** 2014-02-27

**Authors:** Thi-My-Nhung Hoang, Hong-Lien Vu, Ly-Thuy-Tram Le, Chi-Hung Nguyen, Annie Molla

**Affiliations:** 1 INSERM UJF U823 Institut Albert Bonniot, Team 4: Chromatin and Epigenetic, BP 170, 38 042 Grenoble Cedex 9, France; E-Mails: hoangthimynhung@hus.edu.vn (T.-M.-N.H.); honglien162@yahoo.com (H.-L.V.); llttram@gmail.com (L.-T.-T.L.); 2 Faculty of Biology, VNU University of Science, HaNoi 10 000, Vietnam; 3 Department of biology, University of Natural Sciences, Ho Chi Minh 70 000, Vietnam; 4 Department of biology, University of Technology, DaNang 55 000, Vietnam; 5 UMR 176 CNRS-Institut Curie, Bat 110 Centre Universitaire, Orsay 91405, France; E-Mail: Chi.Hung@curie.fr

**Keywords:** aurora, kinase, kinase inhibitor, Gck, Nuak1, Tak-1

## Abstract

Based on *in vitro* assays, we performed a High Throughput Screening (HTS) to identify kinase inhibitors among 10,000 small chemical compounds. In this didactic paper, we describe step-by-step the approach to validate the hits as well as the major pitfalls encountered in the development of active molecules. We propose a decision tree that could be adapted to most *in vitro* HTS.

## 1. Introduction

Aurora kinases are a family of serine/threonine protein kinases that play a key role in mitotic progression [1,2]. In human, three aurora kinases have been identified: A, B and C. Aurora A is initially associated with centrosomes and then with spindle microtubules whereas aurora B is a chromosomal passenger protein travelling from centromeres to microtubules. Aurora A is required for centrosome duplication, entry into mitosis, formation of bipolar spindle and mitotic checkpoint [3,4,5]. Aurora B is essential for chromosome condensation, kinetochore functions, spindle checkpoint activation and cytokinesis completion [2,6,7,8]. Aurora C is poorly described and mostly involved in spermatogenesis [1]. Aurora kinases are over-expressed in many cancers, including primary colon and breast cancers [1,9]. In light of these observations, aurora kinases have emerged as potential “druggable” targets for anti-cancer therapy and many small molecule inhibitors of aurora kinase have been developed [10,11,12,13,14]. Several of these ATP-competitive inhibitors are currently in clinical development. Most of them including benzo[e]pyridoindoles were identified by high throughput screening (HTS) [14]. In the present review we describe step-by-step the HTS performed for selecting aurora kinase inhibitors in the French patrimonial library [14]. The approach followed to validate the hits is outlined and a decision tree is proposed.

## 2. Experimental Section

The protein kinase assay was performed in 20 mM Tris-HCl, 20 mM KCl, 20 mM MgCl_2_, 0.4 µM ATP, 0.4 mM DTT, pH 7.5. Recombinant histone H3 was used as substrate. The reaction was started by the addition of the recombinant kinase domain. After 1 hour of incubation at 37 °C, the remaining ATP was monitored by addition of kinase-Glo^TM^ (Promega, Charbonnières-les-Bains, France) under the conditions suggested by the supplier. Ten minutes later the fluorescence was recorded with a Fluostar Optima (BMG Labtechnologies, Ortenberg, Germany). Staurosporine (0.5 µM) was used as positive control.

Western blotting and immunofluorescence were conducted as already described in [15,16]. Cell proliferation assays were described in [14].

## 3. Results and Discussion

### 3.1. Elaboration of a Test Suitable for HTS

The goal of the screening must be clearly defined. In the search to identify kinase inhibitors two possibilities opened up: either (1) to follow the consumption of ATP or (2) to measure the phosphorylation of a substrate. This decision is crucial since the identified hits will either be (1) ATP-competitive inhibitors or (2) broad inhibitors/activators [17]. The assay has to be very robust with a high Z-factor [18] and easy to scale up. At that step the discussion with the person in charge of the HTS is essential: characteristics of the microplates, quantity of material, time machine and other requirements.

### 3.2. *In Vitro* Aurora Kinase Assay

**Figure 1:**
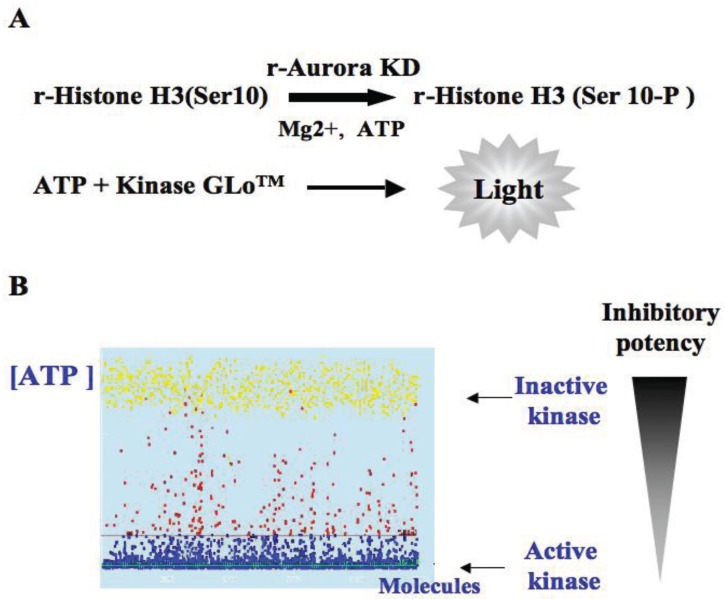
Search for aurora kinase inhibitors. (**A**) Description of the kinase assay: recombinant histone H3 was phosphorylated by the catalytic domain of aurora A under a non-saturating ATP concentration. Remaining ATP was measured by the addition of kinase Glo^TM^; (**B**) Representation of a typical HTS result. When the kinase is inactive the ATP concentration is high. Each point represents a molecule, the yellow points are controls in the presence of staurosporine and blue points the full active kinase in the absence of molecule. The tested compounds are in red. We selected molecules that inhibit aurora kinase by more than 80% at 15 µM.

In a first trial, we developed an ELISA assay in 96-well plates to follow the phosphorylation of histone H3, a well-known substrate of aurora kinases. Unfortunately, the black plastic microplates required for fluorescent reading on the robot were not suitable for coating the basic histone. Finally we decided to measure the consumption of ATP by kinase-Glo^TM^ (Promega, Charbonnières-les-Bains, France) when the recombinant substrate was phosphorylated by a recombinant kinase (Figure 1). The Z-factor of the assay was estimated to be 0.8 [18].

### 3.3. HTS Results

The French patrimonial library is composed of small compounds synthesized by chemists in 29 higher education and research organizations, in France [19]. The primary screening was performed on 10,800 molecules, in triplicate, at a compound concentration of 15 µM. We selected molecules that inhibit aurora kinase by more than 80%. This threshold identified 239 molecules, which represent around 2% of the library. In a second trial, the selected hits were confirmed at the same concentration (15 µM) and tested again at a concentration of 1.5 µM. Approximately 82% of the hits were confirmed to be aurora kinase inhibitors. The next question was: how to deal with these 195 molecules?

### 3.4. Selection of the Active Molecules

The goal of the present study is to identify specific kinase inhibitors. Taking into account that probably several screenings have already been performed on the same library, it is advisable to ask for the removal of all targets previously identified as kinase inhibitors. Before deciding which molecules to select for further studies, you might look to the developed structures. Consider discarding compounds bearing reactive function (e.g., chloride acid or carbaldehyde or Michael acceptor groups) or potentially alkylating agents (e.g., halomethyl substituents) and withdraw those complex molecules for which the synthesis is complicated and costly. Keep in mind that small compounds get generally better through clinical trials than complex molecules [20]. Moreover, it is better to focus on families of molecules highly represented in the panel. Unless you are a chemist, we advise choosing compounds preferentially deposited by an active chemist who could then help for the structure and activity relationship (SAR) study.

Our kinase inhibitor panel was restricted to 127 molecules since other hits were already identified as inhibitors of casein kinase II. Looking at the developed structures of the aurora kinase inhibitors we noticed that two families of molecules were highly represented: flavonoids and benzo[e]pyridoindoles [21] and we focused on them. Note that, at that point, a restricted SAR could already be deduced from the HTS results if several members of a molecule family are identified with different scores [14].

### 3.5. Characterization of the Hits

In most cases, the next step is to use these inhibitors in cells. Several situations may occur that we illustrate by describing pitfalls encountered during the characterisation of the identified aurora kinase inhibitors. The goal was to identify aurora kinase inhibitors with anti-proliferating activity. We checked simultaneously the inhibition of the phosphorylation of histone H3 and the proliferation of cells treated by the molecules.

#### 3.5.1. Results with Flavone Molecules

*In vitro*, we confirmed the full inhibition of histone H3 phosphorylation by the recombinant kinase domain when flavone molecules were added at 15 µM. Two commercial flavones, luteonin and apigenin were also tested and at 1.5 µM, they inhibited histone H3 phosphoylation by 50% and 33% respectively. Unfortunately we did not observe any modification of the level of histone H3 phosphorylation in mitosis and any significant effect on HeLa cells treated for 24 h by these molecules at 1 µM and 5 µM. Although these flavone compounds are efficient towards aurora kinase *in vitro*, they do not target aurora kinase in cell culture. Either these molecules are not permeant enough or they encounter other targets in the cells. In other words, these molecules are aurora kinase inhibitors but are not suitable for cell-based assays.

#### 3.5.2. Results with the Benzo[e]pyridoindole Family

**Figure 2:**
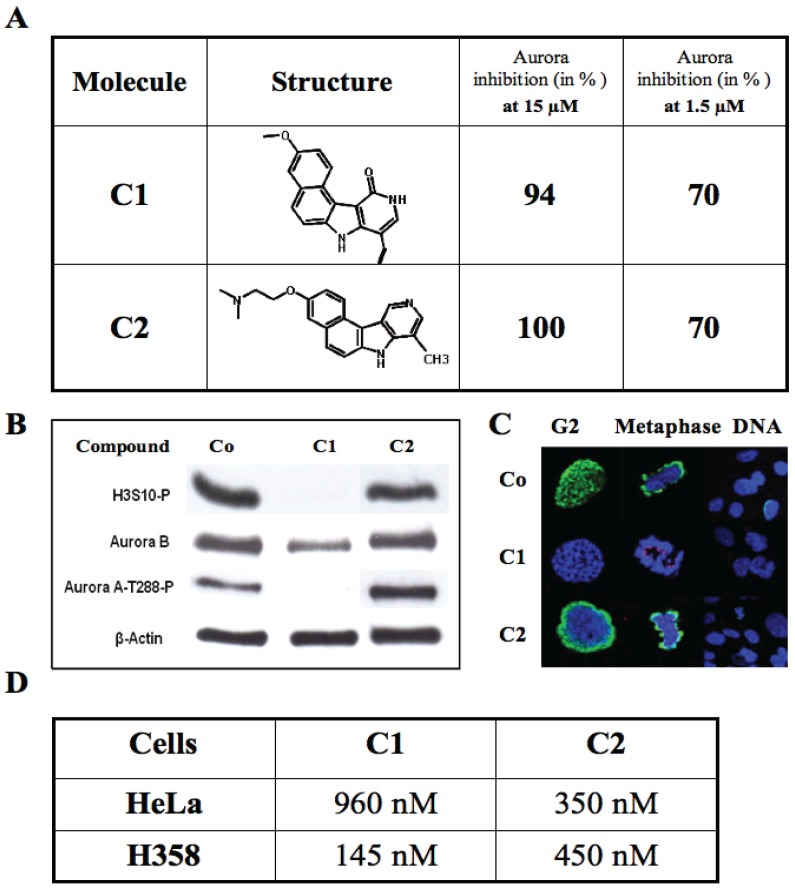
Characterization of the benzo[e]pyridoindoles C1 and C2. (**A**) The structure of C1 and C2 are represented and their efficiency towards the kinase domain of aurora A reported at two different concentrations; (**B**) Immunoblotting on U2OS cells treated by either C1 (1 µM) or C2 (1 µM) or without treatment (Co). ß-actin and aurora B signals are for estimation of the amount of loaded samples and mitotic cells, respectively. The phosphorylation of histone H3 (H3S10-P) reveals aurora kinase-B activity whereas aurora-A-T288-P indicates aurora kinase-A activity; (**C**) Immunofluorescence on U2OS cells treated by either C1 (1 µM) or C2 (1 µM) or without treatment (Co). Histone H3 phosphorylation on Ser10 is shown in green, centromeres are in red and DNA in blue. Cells in G2/prophase and in metaphase were imaged separately as well as in a larger field; (**D**) Cell proliferation assays were conducted for 96 h in the presence of varying C1 and C2 concentrations. IC_50_ values reported in the Table are defined as the concentration that induced a decrease by 50% of the cell population. Results are the mean of triplicate assays.

Analysis of the HTS results reveals that 6% of the hits belong to the benzopyridoindole scaffold deposited by the Curie Institute (France). The most active molecules are C1 described in [14] and C2. *In vitro*, they fully inhibited aurora kinase at 15 µM and decreased kinase activity by 70% at 1.5 µM (Figure 2A). Although both molecules had similar activity *in vitro*, their *in vivo* effects were different. In cells, C1 inhibited both aurora kinases A and B as shown by the decrease of the signals of phospho-Aurora A-T288 and phospho-histone H3-Ser10 respectively (Figure 2B). Moreover it induced mitotic slippage since it decreased the aurora kinase-B signal (Figure 2B). Conversely, C2 was unable to inhibit histone H3 phosphorylation in mitotic cells (Figure 2B,C). Both C1 and C2 exhibited high antiproliferative activities in HeLa and H358 cells with an in cell-IC_50_ of several hundred nanoMolar (Figure 2D).

These two molecules illustrate two situations encountered with the HTS hits: identification of (a) an expected inhibitor like C1 and (b) of a molecule non-targeting the bait in cells, but potentially interesting, like C2.

(a) C1 was found to be an ATP competitive inhibitor, which inhibited *in vitro* aurora kinases at the nanoMolar range [14]. It prevented, *ex vivo*, the phosphorylation of histone H3, and induced mitosis exit without chromosome segregation, known phenomena observed upon aurora B inactivation. This compound was also shown to affect the localization of aurora B, since, in the presence of the inhibitor; the enzyme was delocalized to the whole chromosome [14]. In addition, C1 inhibited the growth of different cell lines derived from different carcinoma. Its IC_50_ for H358 NSCLC (Non Small Cancer Lung Cells), the most sensitive cell line, was 145 nM. Furthermore C1 was found to be efficient towards multicellular tumour spheroid growth [14]. It exhibited minimal toxicity in mice while it had some potency towards aggressive NSCLC tumours. Benzo[e]pyridoindole C1 represents thus a potential lead for the development of aurora kinase inhibitors. Following a SAR study, we designed and synthesised new analogues, bearing an aminoalkyloxy side chain instead of the methoxy group, which may give rise to water-soluble salts. Meanwhile we improved the activity of the hit C1 and we identified new potent hydrosoluble aurora kinase inhibitors that are under *in vivo* evaluation in preclinical animal models of cancer [22]. 

(b) C2 efficiently inhibited *in vitro* the catalytic domain of aurora kinase-A but affected neither aurora A nor B, in cells. C2 did not modify the phosphorylation of histone H3 in G2/M (Figure 2C) and therefore could not even inhibit the basal activity of aurora kinase-B [23]. Apparently, the lack of inhibition is due to the substitution of the oxo group on the pyridine ring by a hydrogen atom [22]. The *in vitro* assay suggested that C2 was an ATP-competitor of kinase domains. Since C2 inhibited the growth of different cells, we searched its potential cell-targets by performing a kinase profiling. The assay conducted by the MRC platform confirmed that C2 was a poor aurora kinase inhibitor (Table 1). Therefore C2 inhibited the catalytic domain of Aurora A (Figure 2A), but is far less active towards the full-length kinase (Table 1). This lack of accessibility to the ATP-binding site explained the lack of efficiency in cells (Figure 2C). At the concentration of 1 µM, only five kinases were inhibited by C2 to arpproximately 50% or more: Gck, TrkA, Nuak1, PhK and Tak1 (Table 1). Several of these kinases were recently implicated in tumour growth and described as potential therapeutic targets [24,25,26]. According to the literature, inhibitors for these kinases are not available and therefore C2 could be a potential lead for future developments.

**Table 1:** Kinase profiling of benzo[e]pyridoindole C2. A kinase profiling was performed *in vitro* in duplicate on 121 recombinant kinases on the MRC platform. (Dundee) The five main targets are listed and the percentage of remaining kinase activity in the presence of C2 (1 µM) is reported. For a clear identification the accession number of each kinase in the databases is indicated. Aurora kinases A and B were poorly affected by C2 (1 µM) since respectively 96 and 86 percent of activity were measured.

Kinases	Accession Number	Remaining Activity (in %)
**GCK** ”Germinal central kinase”	BC047865	32 ± 1
**TrkA** “Tropomyosin Related kinase A”	NM_001007792.1	36 ± 2
**NUAK1–ARK5** “SnF1-like Kinase”	NM_014840	53 ± 2
**PHK** “Phosphorylase b kinase”	X80590	53 ± 7
**Tak1** “Transforming growth factor beta activated kinase”	NM_003188	55 ± 3
**Aurora kinase-B**	NM_004217	86 ± 2
**Aurora kinase-A**	BC027464	96 ± 4

### 3.6. General Discussion

**Figure 3:**
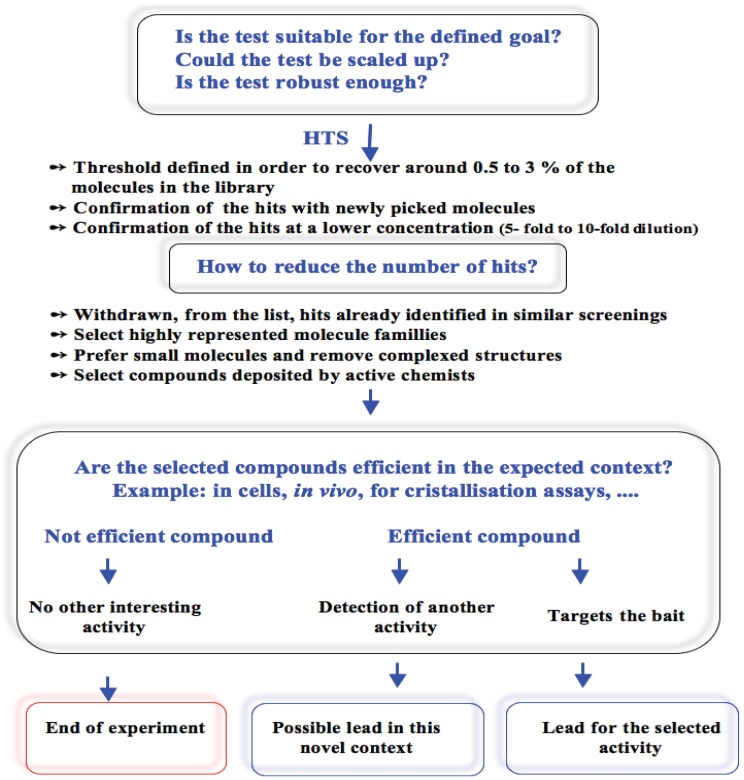
Decision tree for the analysis of HTS hits. The different situations encountered during the search for aurora kinase inhibitors were recorded. A negative reply to any question prevents forward experiments. For each item details are developed in the text through the example of the search for aurora kinase inhibitors.

In conclusion we have described the approach used for the search of aurora kinase inhibitors and we indicated some advice for restraining the hit number. From our experiences three different situations occurred with the HTS hits. In cells, the flavone series had no activity whereas benzo[e]pyridoindole C2 had an unexpected activity and benzo[e]pyridoindole C1 had all requirements we were looking for. C1 and C2 are two leads in the search for new anti-cancer drugs. From these experiences, a decision tree was designed that could be helpful for most *in vitro* HTS (Figure 3).

## 4. Conclusions

In other words, the most challenging step in HTS is not the screen itself, but the next directions. This paper gives advices for succeeding in strategic choices.
